# 6,7-Dimeth­oxy-2,4-di­phenyl­quinoline

**DOI:** 10.1107/S1600536814000725

**Published:** 2014-01-18

**Authors:** M. Prabhuswamy, S. Madan Kumar, T. R. Swaroop, K. S. Rangappa, N. K. Lokanath

**Affiliations:** a Department of Studies in Physics, Manasagangotri, University of Mysore, Mysore 570 006, India; b Department of Studies in Chemistry, Manasagangotri, University of Mysore, Mysore 570 006, India

## Abstract

In the title structure of the title compound, C_23_H_19_NO_2_, two conformationally similar mol­ecules (*A* and *B*) comprise the asymmetric unit. The dihedral angle between phenyl rings bridged by the quinoline moiety are 76.25 (8)° in mol­ecule *A* and 70.39 (9)° in mol­ecule *B*. In the crystal, the independent mol­ecules are connected by C—H⋯O hydrogen bonds and the resulting dimeric aggregates are linked by π–π [inter-centroid distance = 3.7370 (8) Å] and C—H⋯π inter­actions, forming a three-dimensional architecture.

## Related literature   

For general background, and the biological and pharmacological properties of quinoline derivatives, see: Michael (2006 ▶). For a related structure, see: Prabhuswamy *et al.* (2012 ▶).
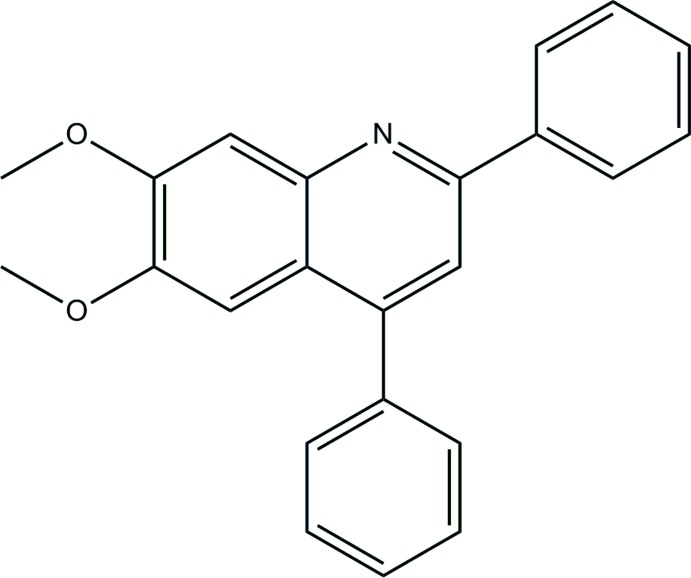


## Experimental   

### 

#### Crystal data  

C_23_H_19_NO_2_
*M* *_r_* = 341.39
Triclinic, 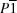

*a* = 8.7092 (3) Å
*b* = 10.5639 (3) Å
*c* = 20.3400 (7) Å
α = 85.678 (1)°
β = 79.397 (1)°
γ = 80.134 (1)°
*V* = 1810.33 (10) Å^3^
*Z* = 4
Cu *K*α radiation
μ = 0.63 mm^−1^
*T* = 296 K
0.23 × 0.21 × 0.14 mm

#### Data collection  

Bruker X8 Proteum diffractometer
Absorption correction: multi-scan (*SAINT-Plus*; Bruker, 2013 ▶) *T*_min_ = 0.868, *T*_max_ = 0.917
21904 measured reflections
5877 independent reflections
5181 reflections with *I* > 2σ(*I*)
*R*_int_ = 0.041

#### Refinement  

*R*[*F* ^2^ > 2σ(*F* ^2^)] = 0.041
*wR*(*F* ^2^) = 0.120
*S* = 1.04
5877 reflections
474 parameters
H-atom parameters constrained
Δρ_max_ = 0.13 e Å^−3^
Δρ_min_ = −0.15 e Å^−3^

### 

Data collection: *APEX2* (Bruker, 2013 ▶); cell refinement: *SAINT-Plus* (Bruker, 2013 ▶); data reduction: *SAINT-Plus*; program(s) used to solve structure: *SHELXS97* (Sheldrick, 2008 ▶); program(s) used to refine structure: *SHELXL97* (Sheldrick, 2008 ▶); molecular graphics: *Mercury* (Macrae *et al.*, 2006 ▶); software used to prepare material for publication: *PLATON* (Spek, 2009 ▶).

## Supplementary Material

Crystal structure: contains datablock(s) global, I. DOI: 10.1107/S1600536814000725/tk5285sup1.cif

Structure factors: contains datablock(s) I. DOI: 10.1107/S1600536814000725/tk5285Isup2.hkl

Supporting information file. DOI: 10.1107/S1600536814000725/tk5285Isup3.cml

CCDC reference:

Additional supporting information:  crystallographic information; 3D view; checkCIF report

## Figures and Tables

**Table 1:** Hydrogen-bond geometry (Å, °) *Cg*6 and *Cg*9 are the centroids of the N10*B*,C7*B*–C9*B*,C11*B*,C12*B* and C21*B*–C26*B* rings, respectively.

*D*—H⋯*A*	*D*—H	H⋯*A*	*D*⋯*A*	*D*—H⋯*A*
C6*A*—H6*A*⋯O19*B* ^i^	0.93	2.58	3.366 (2)	142
C18*B*—H18*F*⋯*Cg*9^ii^	0.96	2.93	3.879 (2)	169
C20*B*—H20*D*⋯*Cg*6^iii^	0.96	2.93	3.59 (18)	127

Symmetry codes: (i) 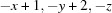; (ii) 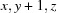; (iii) 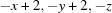.

**Fig. 1.**
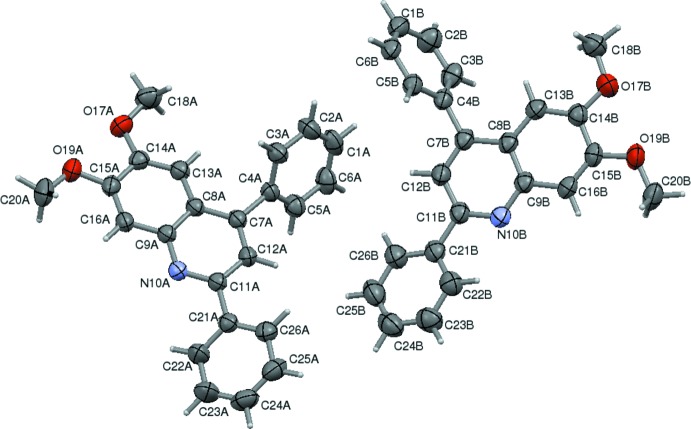
ORTEP view of the title compound with the atom numbering scheme. Displacement ellipsoids for non-H atoms are drawn at 50% probability level.

**Fig. 2.**
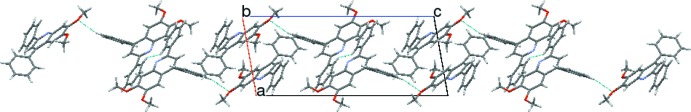
Packing of molecules of the title compound, viewed along the crystallographic b axis. Dotted lines represent C—H···O hydrogen bond interaction.

## supplementary crystallographic information

### 2.1. Synthesis and crystallization

The enaminone (*Z*)-3((3,4-di­meth­oxy­phenyl)­amino)-1,3-di­phenyl­prop-2-en-1-one (5 mmol) was taken in polyphodphoric acid (5 ml) and heated at 140 °C for 5 h. After completion of the reaction (monitored by TLC), the reaction mixture was diluted with water (50 ml). The aqueous layer was extracted with ethyl acetate (3 *X* 20 ml), the combined ethyl acetate­layer was washed with 0.1 N NaOH (2 *X* 25 ml), followed by brine solution (25 ml). The organic layer was then dried over anhydrous sodium sulfate nd concentrated under reduced pressure to afford the crude product 6,7-di­meth­oxy-2,4-di­phenyl­quinoline which was purified by coloumn chromatography over silica gel (60–120 mesh) using a hexane:ethyl acetate mixture (9.5:0.5) as eluent. The pure title compound was crystallized in an ethyl acetate-hexane mixture to obtain pale yellow single crystals.

### 2.2. Refinement

All hydrogen atoms were located geometrically with (C—H = 0.93–0.96) Å and allowed to ride on their parent atoms with *U*_iso_(H) = 1.2*U*_eq_(aromatic C) or 1.5*U*_iso_(methyl C).

### 3. Results and discussion

Quinolines exhibit physico-chemical activities which are useful in the field of pharmaceuticals and agrochemicals. Their derivatives are also present in a wide variety of natural products involved in several biological activities (Michael, 2006). The crystal structure of the title compound is presented here as a part of our on-going structural studies on quinoline derivatives. The asymmetric unit consists of two symmetry-independent title molecules (*A* and *B*) (Fig. 1). The dihedral angle between phenyl ring [C1/C2/C3/C4/C5/C6 (*A/B*)] and quinoline moiety are 60.44 (7)° (*A*) and 56.04 (8)° (*B*). The dihedral angle between phenyl rings bridged by quinoline moiety are 76.25 (8)° (*A*) and 70.39 (9) ° (*B*). Also, the quinoline moiety makes a dihedral angle of 29.14 (8)° (*A*) and 24.64 (8)° (*B*) with phenyl ring C21/C22/C23/C24/C25/C26 (*A/B*). The overall geometry of the title compound is similar to the earlier reported structure of 2-(4-chloro­phenyl)-6-methyl-4-*m*-tolyl quinoline (Prabhuswamy *et al.*, 2012).

The molecules are connected by inter­molecular hydrogen bonds C6A–H6A···O19B (Fig. 2). The crystal structure is further stabilized by π···π inter­actions between Cg(6) and Cg(6)^i^ with a distance of 3.7370 (8) Å [*i*: 1 - *x*, 2 - *y*, -*z*]. The C—H···π inter­actions, C18B–H18F···*Cg*(9) [*x*, 1 + *y*, *z*] and C20B–H20D···*Cg*(6) [2 - *x*, 2 - *y*, *z*], Table 2, link molecules to stabilize the three-dimensional crystal structure.

### Crystal data

**Table:**

C_23_H_19_NO_2_	*Z* = 4
*M**_r_* = 341.39	*F*(000) = 720
Triclinic, *P*1	*D*_x_ = 1.253 Mg m^−^^3^
Hall symbol: -P 1	Cu *K*α radiation, λ = 1.54178 Å
*a* = 8.7092 (3) Å	Cell parameters from 5877 reflections
*b* = 10.5639 (3) Å	θ = 2.2–64.6°
*c* = 20.3400 (7) Å	µ = 0.63 mm^−^^1^
α = 85.678 (1)°	*T* = 296 K
β = 79.397 (1)°	Block, yellow
γ = 80.134 (1)°	0.23 × 0.21 × 0.14 mm
*V* = 1810.33 (10) Å^3^	

### Data collection

**Table:**

Bruker X8 Proteum diffractometer	5877 independent reflections
Radiation source: Bruker MicroStar microfocus rotating anode	5181 reflections with *I* > 2σ(*I*)
Helios multilayer optics monochromator	*R*_int_ = 0.041
Detector resolution: 10.7 pixels mm^-1^	θ_max_ = 64.6°, θ_min_ = 2.2°
φ and ω scans	*h* = −10→9
Absorption correction: multi-scan (*SAINT-Plus*; Bruker, 2013)	*k* = −12→11
*T*_min_ = 0.868, *T*_max_ = 0.917	*l* = −23→23
21904 measured reflections	

### Refinement

**Table:**

Refinement on *F*^2^	Secondary atom site location: difference Fourier map
Least-squares matrix: full	Hydrogen site location: inferred from neighbouring sites
*R*[*F*^2^ > 2σ(*F*^2^)] = 0.041	H-atom parameters constrained
*wR*(*F*^2^) = 0.120	*w* = 1/[σ^2^(*F*_o_^2^) + (0.0647*P*)^2^ + 0.2622*P*] where *P* = (*F*_o_^2^ + 2*F*_c_^2^)/3
*S* = 1.04	(Δ/σ)_max_ < 0.001
5877 reflections	Δρ_max_ = 0.13 e Å^−^^3^
474 parameters	Δρ_min_ = −0.15 e Å^−^^3^
0 restraints	Extinction correction: *SHELXL97* (Sheldrick, 2008), FC^*^=KFC[1+0.001XFC^2^Λ^3^/SIN(2Θ)]^-1/4^
Primary atom site location: structure-invariant direct methods	Extinction coefficient: 0.0037 (4)

### Special details

**Table:**

Experimental. ^1^H NMR (CDCl_3_, 300 MHz): 8.30 (d, J=8.0 Hz, 2H, Ar—H); 7.79 (d, J=8. 0 Hz, 2H, Ar—H); 7.41–7.54 (m, 7H, Ar—H); 7.26 (s, 1H, Ar—H); 7.10 (s,1*H*, Ar—H); 3.91 (s, 3H, OMe); 3.88 (s, 3H, OMe). *M*. P. 122–124 °C (uncorrected)
Geometry. Bond distances, angles *etc*. have been calculated using the rounded fractional coordinates. All su's are estimated from the variances of the (full) variance-covariance matrix. The cell e.s.d.'s are taken into account in the estimation of distances, angles and torsion angles
Refinement. Refinement on *F*^2^ for ALL reflections except those flagged by the user for potential systematic errors. Weighted *R*-factors *wR* and all goodnesses of fit *S* are based on *F*^2^, conventional *R*-factors *R* are based on *F*, with *F* set to zero for negative *F*^2^. The observed criterion of *F*^2^ > σ(*F*^2^) is used only for calculating -*R*-factor-obs *etc*. and is not relevant to the choice of reflections for refinement. *R*-factors based on *F*^2^ are statistically about twice as large as those based on *F*, and *R*-factors based on ALL data will be even larger.

### Fractional atomic coordinates and isotropic or equivalent isotropic displacement parameters (Å^2^)

**Table:**

	*x*	*y*	*z*	*U*_iso_*/*U*_eq_	
O17A	−0.05130 (13)	0.68563 (10)	0.59999 (5)	0.0604 (3)	
O19A	0.05651 (14)	0.49729 (10)	0.67519 (5)	0.0620 (4)	
N10A	0.42927 (14)	0.31063 (10)	0.49004 (5)	0.0452 (4)	
C1A	0.27076 (19)	0.86258 (16)	0.27831 (9)	0.0637 (6)	
C2A	0.3218 (2)	0.87201 (15)	0.33749 (9)	0.0650 (6)	
C3A	0.3444 (2)	0.76504 (14)	0.38049 (8)	0.0566 (5)	
C4A	0.31857 (16)	0.64666 (13)	0.36342 (6)	0.0447 (4)	
C5A	0.26951 (18)	0.63793 (15)	0.30320 (7)	0.0544 (5)	
C6A	0.2447 (2)	0.74626 (17)	0.26104 (8)	0.0629 (6)	
C7A	0.35107 (16)	0.52976 (12)	0.40763 (6)	0.0432 (4)	
C8A	0.26898 (16)	0.52108 (12)	0.47472 (6)	0.0418 (4)	
C9A	0.31638 (16)	0.40926 (12)	0.51407 (6)	0.0426 (4)	
C11A	0.50062 (16)	0.31963 (12)	0.42656 (6)	0.0433 (4)	
C12A	0.46396 (17)	0.42894 (12)	0.38462 (6)	0.0450 (4)	
C13A	0.14306 (17)	0.61463 (13)	0.50322 (7)	0.0463 (4)	
C14A	0.07132 (17)	0.60191 (13)	0.56843 (7)	0.0471 (4)	
C15A	0.12742 (17)	0.49384 (14)	0.60936 (7)	0.0482 (4)	
C16A	0.24375 (17)	0.39985 (13)	0.58243 (7)	0.0472 (4)	
C18A	−0.1033 (2)	0.79914 (17)	0.56278 (10)	0.0718 (6)	
C20A	0.1173 (3)	0.39846 (19)	0.71948 (8)	0.0763 (7)	
C21A	0.62097 (17)	0.20846 (13)	0.40190 (7)	0.0465 (4)	
C22A	0.6062 (2)	0.08659 (15)	0.42997 (9)	0.0683 (6)	
C23A	0.7163 (3)	−0.01903 (17)	0.40975 (10)	0.0810 (7)	
C24A	0.8455 (2)	−0.00526 (18)	0.36108 (10)	0.0749 (7)	
C25A	0.8608 (2)	0.11475 (17)	0.33214 (9)	0.0677 (6)	
C26A	0.74891 (19)	0.22035 (15)	0.35167 (7)	0.0547 (5)	
O17B	0.76986 (15)	1.31226 (11)	−0.07274 (6)	0.0696 (4)	
O19B	0.95184 (14)	1.13055 (11)	−0.13693 (5)	0.0644 (4)	
N10B	0.79196 (15)	0.81305 (12)	0.02976 (6)	0.0525 (4)	
C1B	0.2756 (2)	1.26810 (16)	0.24162 (8)	0.0623 (5)	
C2B	0.2546 (2)	1.26622 (18)	0.17618 (9)	0.0685 (6)	
C3B	0.35796 (19)	1.18455 (17)	0.13196 (8)	0.0610 (5)	
C4B	0.48281 (17)	1.10097 (14)	0.15279 (7)	0.0485 (4)	
C5B	0.50382 (18)	1.10581 (14)	0.21888 (7)	0.0518 (5)	
C6B	0.4010 (2)	1.18922 (15)	0.26210 (7)	0.0575 (5)	
C7B	0.58853 (18)	1.00442 (14)	0.10822 (7)	0.0502 (5)	
C8B	0.67728 (17)	1.03974 (14)	0.04528 (7)	0.0481 (4)	
C9B	0.77830 (17)	0.93921 (14)	0.00888 (7)	0.0489 (4)	
C11B	0.70799 (18)	0.78223 (14)	0.08849 (7)	0.0521 (5)	
C12B	0.60666 (19)	0.87656 (15)	0.12840 (7)	0.0554 (5)	
C13B	0.67477 (18)	1.16778 (15)	0.01843 (7)	0.0521 (5)	
C14B	0.76529 (18)	1.19406 (14)	−0.04178 (7)	0.0528 (5)	
C15B	0.86679 (18)	1.09210 (15)	−0.07799 (6)	0.0511 (5)	
C16B	0.87288 (18)	0.96897 (15)	−0.05314 (7)	0.0513 (5)	
C18B	0.6658 (3)	1.41683 (17)	−0.04165 (10)	0.0771 (7)	
C20B	1.0476 (2)	1.03192 (19)	−0.17638 (8)	0.0703 (6)	
C21B	0.72866 (19)	0.64325 (15)	0.10957 (7)	0.0535 (5)	
C22B	0.8667 (2)	0.56210 (16)	0.08426 (8)	0.0636 (6)	
C23B	0.8868 (3)	0.43197 (18)	0.10154 (10)	0.0754 (7)	
C24B	0.7698 (3)	0.38066 (19)	0.14503 (10)	0.0789 (7)	
C25B	0.6340 (3)	0.45959 (19)	0.17076 (10)	0.0788 (7)	
C26B	0.6125 (2)	0.58966 (17)	0.15338 (9)	0.0673 (6)	
H1A	0.25400	0.93510	0.25000	0.0760*	
H2A	0.34120	0.95080	0.34880	0.0780*	
H3A	0.37710	0.77260	0.42090	0.0680*	
H5A	0.25310	0.55900	0.29090	0.0650*	
H6A	0.21020	0.73980	0.22100	0.0760*	
H12A	0.51740	0.43250	0.34070	0.0540*	
H13A	0.10840	0.68620	0.47710	0.0560*	
H16A	0.27630	0.32840	0.60910	0.0570*	
H18A	−0.01720	0.84660	0.54900	0.1080*	
H18B	−0.18870	0.85120	0.59020	0.1080*	
H18C	−0.13900	0.77630	0.52400	0.1080*	
H20A	0.10070	0.31670	0.70690	0.1140*	
H20B	0.06370	0.41390	0.76450	0.1140*	
H20C	0.22860	0.39810	0.71690	0.1140*	
H22A	0.51990	0.07600	0.46320	0.0820*	
H23A	0.70330	−0.10000	0.42900	0.0970*	
H24A	0.92140	−0.07620	0.34790	0.0900*	
H25A	0.94750	0.12490	0.29900	0.0810*	
H26A	0.75970	0.30050	0.33080	0.0660*	
H1B	0.20540	1.32230	0.27140	0.0750*	
H2B	0.17030	1.32030	0.16160	0.0820*	
H3B	0.34350	1.18570	0.08770	0.0730*	
H5B	0.58790	1.05230	0.23400	0.0620*	
H6B	0.41740	1.19170	0.30590	0.0690*	
H12B	0.55070	0.85160	0.16940	0.0660*	
H13B	0.61050	1.23490	0.04220	0.0620*	
H16B	0.93980	0.90330	−0.07700	0.0620*	
H18D	0.55880	1.40100	−0.03650	0.1160*	
H18E	0.67680	1.49410	−0.06890	0.1160*	
H18F	0.69090	1.42650	0.00150	0.1160*	
H20D	1.12500	0.98500	−0.15180	0.1060*	
H20E	1.10030	1.06940	−0.21690	0.1060*	
H20F	0.98250	0.97450	−0.18720	0.1060*	
H22B	0.94680	0.59590	0.05520	0.0760*	
H23B	0.97950	0.37890	0.08380	0.0910*	
H24B	0.78310	0.29320	0.15680	0.0950*	
H25B	0.55510	0.42540	0.20030	0.0950*	
H26B	0.51920	0.64180	0.17120	0.0810*	

### Atomic displacement parameters (Å^2^)

**Table:**

	*U*^11^	*U*^22^	*U*^33^	*U*^12^	*U*^13^	*U*^23^
O17A	0.0577 (6)	0.0581 (6)	0.0594 (6)	−0.0022 (5)	0.0021 (5)	−0.0115 (5)
O19A	0.0754 (7)	0.0645 (7)	0.0415 (5)	−0.0149 (5)	0.0056 (5)	−0.0036 (5)
N10A	0.0557 (7)	0.0386 (6)	0.0404 (6)	−0.0073 (5)	−0.0070 (5)	0.0002 (4)
C1A	0.0562 (9)	0.0581 (10)	0.0674 (10)	−0.0009 (7)	−0.0050 (7)	0.0245 (8)
C2A	0.0771 (11)	0.0421 (8)	0.0718 (11)	−0.0082 (7)	−0.0086 (9)	0.0095 (7)
C3A	0.0727 (10)	0.0434 (8)	0.0538 (8)	−0.0091 (7)	−0.0143 (7)	0.0038 (6)
C4A	0.0474 (7)	0.0417 (7)	0.0419 (7)	−0.0046 (6)	−0.0048 (6)	0.0043 (5)
C5A	0.0597 (9)	0.0545 (8)	0.0499 (8)	−0.0100 (7)	−0.0139 (7)	0.0040 (6)
C6A	0.0616 (10)	0.0744 (11)	0.0506 (8)	−0.0070 (8)	−0.0156 (7)	0.0165 (7)
C7A	0.0515 (8)	0.0382 (7)	0.0410 (7)	−0.0103 (6)	−0.0097 (6)	0.0015 (5)
C8A	0.0493 (7)	0.0375 (7)	0.0402 (7)	−0.0112 (6)	−0.0086 (6)	0.0001 (5)
C9A	0.0500 (7)	0.0379 (7)	0.0406 (7)	−0.0100 (6)	−0.0076 (6)	−0.0011 (5)
C11A	0.0506 (8)	0.0384 (7)	0.0413 (7)	−0.0088 (6)	−0.0081 (6)	−0.0013 (5)
C12A	0.0547 (8)	0.0417 (7)	0.0376 (6)	−0.0089 (6)	−0.0055 (6)	0.0015 (5)
C13A	0.0511 (8)	0.0413 (7)	0.0468 (7)	−0.0079 (6)	−0.0092 (6)	−0.0008 (6)
C14A	0.0477 (7)	0.0456 (7)	0.0486 (7)	−0.0104 (6)	−0.0044 (6)	−0.0088 (6)
C15A	0.0546 (8)	0.0507 (8)	0.0406 (7)	−0.0182 (6)	−0.0011 (6)	−0.0051 (6)
C16A	0.0594 (8)	0.0427 (7)	0.0400 (7)	−0.0134 (6)	−0.0066 (6)	0.0020 (5)
C18A	0.0694 (11)	0.0617 (10)	0.0766 (11)	0.0099 (8)	−0.0084 (9)	−0.0116 (8)
C20A	0.0955 (14)	0.0883 (13)	0.0417 (8)	−0.0177 (11)	−0.0042 (8)	0.0056 (8)
C21A	0.0546 (8)	0.0430 (7)	0.0423 (7)	−0.0059 (6)	−0.0108 (6)	−0.0028 (5)
C22A	0.0830 (12)	0.0456 (8)	0.0630 (10)	0.0028 (8)	0.0065 (8)	0.0058 (7)
C23A	0.1048 (15)	0.0467 (9)	0.0766 (12)	0.0104 (9)	−0.0001 (11)	0.0018 (8)
C24A	0.0804 (12)	0.0610 (11)	0.0758 (11)	0.0148 (9)	−0.0112 (10)	−0.0211 (9)
C25A	0.0626 (10)	0.0716 (11)	0.0658 (10)	−0.0066 (8)	0.0015 (8)	−0.0237 (8)
C26A	0.0613 (9)	0.0506 (8)	0.0528 (8)	−0.0126 (7)	−0.0052 (7)	−0.0098 (6)
O17B	0.0852 (8)	0.0573 (7)	0.0588 (6)	−0.0059 (6)	−0.0026 (6)	0.0086 (5)
O19B	0.0722 (7)	0.0734 (7)	0.0400 (5)	−0.0069 (6)	0.0010 (5)	0.0085 (5)
N10B	0.0612 (7)	0.0534 (7)	0.0430 (6)	−0.0122 (6)	−0.0058 (5)	−0.0045 (5)
C1B	0.0676 (10)	0.0576 (9)	0.0588 (9)	−0.0155 (8)	0.0063 (8)	−0.0137 (7)
C2B	0.0567 (10)	0.0757 (11)	0.0703 (11)	−0.0029 (8)	−0.0103 (8)	−0.0054 (8)
C3B	0.0589 (9)	0.0794 (11)	0.0476 (8)	−0.0146 (8)	−0.0115 (7)	−0.0086 (7)
C4B	0.0525 (8)	0.0540 (8)	0.0411 (7)	−0.0192 (7)	−0.0023 (6)	−0.0062 (6)
C5B	0.0585 (9)	0.0564 (8)	0.0411 (7)	−0.0144 (7)	−0.0059 (6)	−0.0019 (6)
C6B	0.0735 (10)	0.0595 (9)	0.0413 (7)	−0.0213 (8)	−0.0021 (7)	−0.0082 (6)
C7B	0.0544 (8)	0.0577 (9)	0.0408 (7)	−0.0171 (7)	−0.0040 (6)	−0.0086 (6)
C8B	0.0535 (8)	0.0546 (8)	0.0385 (7)	−0.0145 (6)	−0.0068 (6)	−0.0068 (6)
C9B	0.0553 (8)	0.0548 (8)	0.0389 (7)	−0.0145 (7)	−0.0078 (6)	−0.0048 (6)
C11B	0.0587 (9)	0.0566 (8)	0.0432 (7)	−0.0179 (7)	−0.0060 (6)	−0.0040 (6)
C12B	0.0649 (9)	0.0583 (9)	0.0433 (7)	−0.0207 (7)	0.0007 (7)	−0.0049 (6)
C13B	0.0588 (9)	0.0538 (8)	0.0437 (7)	−0.0102 (7)	−0.0060 (6)	−0.0078 (6)
C14B	0.0603 (9)	0.0550 (8)	0.0439 (7)	−0.0113 (7)	−0.0110 (6)	0.0023 (6)
C15B	0.0543 (8)	0.0646 (9)	0.0345 (7)	−0.0120 (7)	−0.0074 (6)	0.0015 (6)
C16B	0.0572 (9)	0.0587 (9)	0.0372 (7)	−0.0086 (7)	−0.0052 (6)	−0.0059 (6)
C18B	0.0986 (14)	0.0578 (10)	0.0701 (11)	−0.0016 (9)	−0.0152 (10)	0.0032 (8)
C20B	0.0663 (10)	0.0897 (12)	0.0443 (8)	0.0012 (9)	0.0026 (7)	0.0036 (8)
C21B	0.0635 (9)	0.0556 (8)	0.0450 (7)	−0.0174 (7)	−0.0118 (7)	−0.0025 (6)
C22B	0.0667 (10)	0.0640 (10)	0.0600 (9)	−0.0118 (8)	−0.0119 (8)	0.0023 (7)
C23B	0.0835 (13)	0.0639 (11)	0.0776 (12)	−0.0013 (9)	−0.0231 (10)	0.0030 (9)
C24B	0.1077 (16)	0.0585 (10)	0.0750 (12)	−0.0177 (11)	−0.0284 (11)	0.0099 (9)
C25B	0.0996 (15)	0.0705 (12)	0.0712 (11)	−0.0360 (11)	−0.0132 (10)	0.0125 (9)
C26B	0.0750 (11)	0.0630 (10)	0.0634 (10)	−0.0205 (9)	−0.0031 (8)	−0.0001 (8)

### Geometric parameters (Å, º)

**Table:**

O17A—C14A	1.3581 (18)	C20A—H20C	0.9600
O17A—C18A	1.421 (2)	C22A—H22A	0.9300
O19A—C15A	1.3678 (17)	C23A—H23A	0.9300
O19A—C20A	1.423 (2)	C24A—H24A	0.9300
O17B—C18B	1.412 (2)	C25A—H25A	0.9300
O17B—C14B	1.3589 (19)	C26A—H26A	0.9300
O19B—C20B	1.417 (2)	C1B—C2B	1.379 (2)
O19B—C15B	1.3615 (17)	C1B—C6B	1.365 (2)
N10A—C11A	1.3300 (16)	C2B—C3B	1.384 (2)
N10A—C9A	1.3565 (17)	C3B—C4B	1.388 (2)
N10B—C11B	1.3302 (19)	C4B—C5B	1.395 (2)
N10B—C9B	1.3600 (19)	C4B—C7B	1.490 (2)
C1A—C6A	1.369 (2)	C5B—C6B	1.381 (2)
C1A—C2A	1.374 (3)	C7B—C8B	1.428 (2)
C2A—C3A	1.385 (2)	C7B—C12B	1.373 (2)
C3A—C4A	1.388 (2)	C8B—C9B	1.417 (2)
C4A—C5A	1.3837 (19)	C8B—C13B	1.418 (2)
C4A—C7A	1.4901 (18)	C9B—C16B	1.420 (2)
C5A—C6A	1.388 (2)	C11B—C12B	1.410 (2)
C7A—C8A	1.4235 (17)	C11B—C21B	1.488 (2)
C7A—C12A	1.3664 (19)	C13B—C14B	1.366 (2)
C8A—C13A	1.4129 (19)	C14B—C15B	1.428 (2)
C8A—C9A	1.4207 (18)	C15B—C16B	1.356 (2)
C9A—C16A	1.4214 (19)	C21B—C22B	1.390 (2)
C11A—C12A	1.4101 (18)	C21B—C26B	1.388 (2)
C11A—C21A	1.4842 (19)	C22B—C23B	1.383 (3)
C13A—C14A	1.366 (2)	C23B—C24B	1.378 (3)
C14A—C15A	1.432 (2)	C24B—C25B	1.367 (3)
C15A—C16A	1.358 (2)	C25B—C26B	1.382 (3)
C21A—C22A	1.385 (2)	C1B—H1B	0.9300
C21A—C26A	1.382 (2)	C2B—H2B	0.9300
C22A—C23A	1.375 (3)	C3B—H3B	0.9300
C23A—C24A	1.375 (3)	C5B—H5B	0.9300
C24A—C25A	1.374 (3)	C6B—H6B	0.9300
C25A—C26A	1.380 (2)	C12B—H12B	0.9300
C1A—H1A	0.9300	C13B—H13B	0.9300
C2A—H2A	0.9300	C16B—H16B	0.9300
C3A—H3A	0.9300	C18B—H18D	0.9600
C5A—H5A	0.9300	C18B—H18E	0.9600
C6A—H6A	0.9300	C18B—H18F	0.9600
C12A—H12A	0.9300	C20B—H20D	0.9600
C13A—H13A	0.9300	C20B—H20E	0.9600
C16A—H16A	0.9300	C20B—H20F	0.9600
C18A—H18C	0.9600	C22B—H22B	0.9300
C18A—H18B	0.9600	C23B—H23B	0.9300
C18A—H18A	0.9600	C24B—H24B	0.9300
C20A—H20A	0.9600	C25B—H25B	0.9300
C20A—H20B	0.9600	C26B—H26B	0.9300
			
O17A···O19A	2.5709 (15)	C26B···H12B	2.7600
O17A···C25A^i^	3.404 (2)	C26B···H18D^iii^	3.0200
O17A···C26A^i^	3.299 (2)	H1A···C5B	3.0100
O17B···O19B	2.5446 (16)	H1B···O19A^iv^	2.8200
O19A···O17A	2.5709 (15)	H1B···O17A^iv^	2.7100
O19A···C5A^ii^	3.336 (2)	H2A···H18B^iv^	2.5500
O19B···C6A^iii^	3.366 (2)	H2A···C6B	3.0300
O19B···O17B	2.5446 (16)	H2B···C23B^ix^	2.9800
O17A···H1B^iv^	2.7100	H2B···H23B^ix^	2.4700
O17B···H22B^v^	2.8900	H3A···C8A	3.0300
O19A···H5A^ii^	2.8100	H3A···C13A	3.0400
O19A···H26A^i^	2.8600	H3A···N10A^i^	2.7000
O19A···H1B^iv^	2.8200	H3B···C16B^iii^	2.9000
O19B···H6A^iii^	2.5800	H3B···C13B	2.9500
N10A···H22A	2.5400	H3B···C8B	3.0500
N10A···H23A^vi^	2.9400	H3B···C9B^iii^	2.8900
N10A···H3A^i^	2.7000	H3B···H13B	2.4700
N10A···H18C^ii^	2.9000	H3B···N10B^iii^	2.8500
N10B···H22B	2.5200	H5A···O19A^ii^	2.8100
N10B···H3B^iii^	2.8500	H5A···C12A	2.9900
C1A···C5B	3.550 (2)	H5B···C12B	2.9100
C3A···C13A	3.252 (2)	H6A···O19B^iii^	2.5800
C3B···C13B	3.244 (2)	H6B···C21A^xii^	2.9000
C3B···C16B^iii^	3.455 (2)	H12A···C26A	2.7700
C5A···O19A^ii^	3.336 (2)	H12A···H26A	2.3100
C5A···C20A^ii^	3.571 (3)	H12A···C5A	2.9500
C5B···C1A	3.550 (2)	H12B···C1A	2.9700
C6A···O19B^iii^	3.366 (2)	H12B···H26B	2.2800
C9B···C15B^v^	3.580 (2)	H12B···C26B	2.7600
C12A···C16A^i^	3.548 (2)	H12B···C5B	2.8800
C13A···C3A	3.252 (2)	H13A···C18A	2.5000
C13B···C3B	3.244 (2)	H13A···C4A	2.6800
C14A···C26A^i^	3.320 (2)	H13A···C3A	2.7600
C15A···C26A^i^	3.563 (2)	H13A···H18A	2.3100
C15B···C9B^v^	3.580 (2)	H13A···H18C	2.2700
C16A···C12A^i^	3.548 (2)	H13B···C18B	2.5200
C16B···C3B^iii^	3.455 (2)	H13B···C3B	2.6900
C16B···C16B^v^	3.533 (2)	H13B···C4B	2.7300
C18A···C25A^i^	3.531 (3)	H13B···H18D	2.3300
C18B···C18B^vii^	3.348 (3)	H13B···H18F	2.3000
C20A···C5A^ii^	3.571 (3)	H13B···H3B	2.4700
C20B···C25A^viii^	3.530 (2)	H16A···H23A^vi^	2.5600
C25A···C20B^viii^	3.530 (2)	H16A···H20A	2.2800
C25A···O17A^i^	3.404 (2)	H16A···H20C	2.3100
C25A···C18A^i^	3.531 (3)	H16A···C20A	2.5000
C26A···O17A^i^	3.299 (2)	H16B···H20F	2.2900
C26A···C15A^i^	3.563 (2)	H16B···H20D	2.2400
C26A···C14A^i^	3.320 (2)	H16B···C20B	2.4800
C1A···H12B	2.9700	H18A···C25A^i^	3.0700
C1A···H24A^ix^	3.0900	H18A···C13A	2.7400
C3A···H13A	2.7600	H18A···H13A	2.3100
C3B···H13B	2.6900	H18B···H2A^iv^	2.5500
C4A···H13A	2.6800	H18C···N10A^ii^	2.9000
C4B···H13B	2.7300	H18C···C13A	2.7200
C5A···H12A	2.9500	H18C···H13A	2.2700
C5B···H12B	2.8800	H18C···C9A^ii^	2.9200
C5B···H1A	3.0100	H18D···C13B	2.7400
C6A···H26B	2.8400	H18D···H13B	2.3300
C6B···H2A	3.0300	H18D···C18B^vii^	2.8400
C7B···H20D^v^	2.8200	H18D···C26B^iii^	3.0200
C8A···H3A	3.0300	H18F···H13B	2.3000
C8B···H3B	3.0500	H18F···C23B^xii^	2.9000
C8B···H20D^v^	2.9800	H18F···C13B	2.7500
C9A···H18C^ii^	2.9200	H18F···C22B^xii^	3.0500
C9B···H3B^iii^	2.8900	H20A···C16A	2.7600
C12A···H5A	2.9900	H20A···H16A	2.2800
C12A···H26A	2.7700	H20C···C16A	2.7100
C12B···H5B	2.9100	H20C···H16A	2.3100
C12B···H20D^v^	3.0800	H20D···C16B	2.7100
C12B···H26B	2.7500	H20D···C8B^v^	2.9800
C13A···H18A	2.7400	H20D···C12B^v^	3.0800
C13A···H18C	2.7200	H20D···C7B^v^	2.8200
C13A···H3A	3.0400	H20D···H16B	2.2400
C13B···H18D	2.7400	H20E···C25A^viii^	3.0900
C13B···H3B	2.9500	H20E···C24A^viii^	2.9900
C13B···H18F	2.7500	H20F···H25A^viii^	2.5100
C14A···H26A^i^	3.0600	H20F···C16B	2.7200
C15A···H26A^i^	2.9600	H20F···H16B	2.2900
C16A···H20A	2.7600	H22A···N10A	2.5400
C16A···H20C	2.7100	H22A···C22A^vi^	2.8400
C16B···H20F	2.7200	H22A···C23A^vi^	3.0800
C16B···H20D	2.7100	H22A···H22A^vi^	2.1400
C16B···H3B^iii^	2.9000	H22B···N10B	2.5200
C18A···H13A	2.5000	H22B···O17B^v^	2.8900
C18B···H18D^vii^	2.8400	H23A···N10A^vi^	2.9400
C18B···H13B	2.5200	H23A···H16A^vi^	2.5600
C20A···H16A	2.5000	H23B···H2B^xi^	2.4700
C20B···H16B	2.4800	H24A···C1A^xi^	3.0900
C20B···H25A^viii^	3.0900	H25A···H20F^viii^	2.5100
C21A···H6B^x^	2.9000	H25A···C20B^viii^	3.0900
C22A···H22A^vi^	2.8400	H26A···O19A^i^	2.8600
C22B···H18F^x^	3.0500	H26A···C14A^i^	3.0600
C23A···H22A^vi^	3.0800	H26A···C15A^i^	2.9600
C23B···H2B^xi^	2.9800	H26A···H12A	2.3100
C23B···H18F^x^	2.9000	H26A···C12A	2.7700
C24A···H20E^viii^	2.9900	H26B···C6A	2.8400
C25A···H18A^i^	3.0700	H26B···C12B	2.7500
C25A···H20E^viii^	3.0900	H26B···H12B	2.2800
C26A···H12A	2.7700		
			
C14A—O17A—C18A	116.90 (12)	C26A—C25A—H25A	120.00
C15A—O19A—C20A	116.88 (13)	C21A—C26A—H26A	120.00
C14B—O17B—C18B	117.15 (13)	C25A—C26A—H26A	120.00
C15B—O19B—C20B	116.43 (13)	C2B—C1B—C6B	119.05 (15)
C9A—N10A—C11A	118.34 (11)	C1B—C2B—C3B	120.60 (16)
C9B—N10B—C11B	118.14 (13)	C2B—C3B—C4B	120.85 (15)
C2A—C1A—C6A	119.87 (16)	C3B—C4B—C5B	117.72 (14)
C1A—C2A—C3A	120.45 (15)	C3B—C4B—C7B	122.35 (13)
C2A—C3A—C4A	120.13 (15)	C5B—C4B—C7B	119.88 (13)
C3A—C4A—C5A	118.89 (13)	C4B—C5B—C6B	120.68 (14)
C3A—C4A—C7A	120.44 (12)	C1B—C6B—C5B	121.06 (14)
C5A—C4A—C7A	120.59 (12)	C4B—C7B—C8B	122.49 (13)
C4A—C5A—C6A	120.44 (14)	C4B—C7B—C12B	119.72 (13)
C1A—C6A—C5A	120.20 (15)	C8B—C7B—C12B	117.77 (13)
C4A—C7A—C8A	121.38 (11)	C7B—C8B—C9B	116.93 (13)
C4A—C7A—C12A	120.15 (11)	C7B—C8B—C13B	124.48 (13)
C8A—C7A—C12A	118.46 (11)	C9B—C8B—C13B	118.55 (13)
C7A—C8A—C9A	116.90 (12)	N10B—C9B—C8B	123.93 (13)
C9A—C8A—C13A	119.02 (11)	N10B—C9B—C16B	116.62 (13)
C7A—C8A—C13A	124.07 (12)	C8B—C9B—C16B	119.45 (13)
N10A—C9A—C8A	123.41 (11)	N10B—C11B—C12B	121.56 (13)
C8A—C9A—C16A	118.80 (12)	N10B—C11B—C21B	116.13 (13)
N10A—C9A—C16A	117.79 (11)	C12B—C11B—C21B	122.31 (13)
N10A—C11A—C21A	116.59 (11)	C7B—C12B—C11B	121.65 (13)
N10A—C11A—C12A	121.88 (12)	C8B—C13B—C14B	121.01 (14)
C12A—C11A—C21A	121.53 (11)	O17B—C14B—C13B	125.99 (14)
C7A—C12A—C11A	120.95 (11)	O17B—C14B—C15B	114.04 (13)
C8A—C13A—C14A	121.20 (13)	C13B—C14B—C15B	119.97 (13)
C13A—C14A—C15A	119.55 (13)	O19B—C15B—C14B	114.45 (13)
O17A—C14A—C13A	125.51 (13)	O19B—C15B—C16B	125.29 (14)
O17A—C14A—C15A	114.93 (12)	C14B—C15B—C16B	120.26 (13)
O19A—C15A—C14A	114.37 (12)	C9B—C16B—C15B	120.74 (14)
O19A—C15A—C16A	125.30 (13)	C11B—C21B—C22B	119.95 (14)
C14A—C15A—C16A	120.32 (13)	C11B—C21B—C26B	122.21 (15)
C9A—C16A—C15A	120.86 (13)	C22B—C21B—C26B	117.83 (15)
C22A—C21A—C26A	117.74 (14)	C21B—C22B—C23B	121.05 (17)
C11A—C21A—C22A	119.28 (13)	C22B—C23B—C24B	120.2 (2)
C11A—C21A—C26A	122.99 (13)	C23B—C24B—C25B	119.37 (19)
C21A—C22A—C23A	121.45 (17)	C24B—C25B—C26B	120.8 (2)
C22A—C23A—C24A	120.20 (17)	C21B—C26B—C25B	120.75 (18)
C23A—C24A—C25A	119.05 (17)	C2B—C1B—H1B	121.00
C24A—C25A—C26A	120.71 (16)	C6B—C1B—H1B	120.00
C21A—C26A—C25A	120.82 (14)	C1B—C2B—H2B	120.00
C2A—C1A—H1A	120.00	C3B—C2B—H2B	120.00
C6A—C1A—H1A	120.00	C2B—C3B—H3B	120.00
C3A—C2A—H2A	120.00	C4B—C3B—H3B	120.00
C1A—C2A—H2A	120.00	C4B—C5B—H5B	120.00
C2A—C3A—H3A	120.00	C6B—C5B—H5B	120.00
C4A—C3A—H3A	120.00	C1B—C6B—H6B	119.00
C4A—C5A—H5A	120.00	C5B—C6B—H6B	120.00
C6A—C5A—H5A	120.00	C7B—C12B—H12B	119.00
C1A—C6A—H6A	120.00	C11B—C12B—H12B	119.00
C5A—C6A—H6A	120.00	C8B—C13B—H13B	119.00
C7A—C12A—H12A	120.00	C14B—C13B—H13B	120.00
C11A—C12A—H12A	120.00	C9B—C16B—H16B	120.00
C8A—C13A—H13A	119.00	C15B—C16B—H16B	120.00
C14A—C13A—H13A	119.00	O17B—C18B—H18D	109.00
C9A—C16A—H16A	120.00	O17B—C18B—H18E	109.00
C15A—C16A—H16A	120.00	O17B—C18B—H18F	110.00
H18A—C18A—H18B	109.00	H18D—C18B—H18E	109.00
H18A—C18A—H18C	110.00	H18D—C18B—H18F	110.00
H18B—C18A—H18C	109.00	H18E—C18B—H18F	109.00
O17A—C18A—H18C	109.00	O19B—C20B—H20D	110.00
O17A—C18A—H18A	109.00	O19B—C20B—H20E	109.00
O17A—C18A—H18B	109.00	O19B—C20B—H20F	110.00
O19A—C20A—H20A	109.00	H20D—C20B—H20E	109.00
H20A—C20A—H20C	109.00	H20D—C20B—H20F	109.00
H20B—C20A—H20C	109.00	H20E—C20B—H20F	109.00
O19A—C20A—H20B	110.00	C21B—C22B—H22B	119.00
O19A—C20A—H20C	109.00	C23B—C22B—H22B	120.00
H20A—C20A—H20B	109.00	C22B—C23B—H23B	120.00
C21A—C22A—H22A	119.00	C24B—C23B—H23B	120.00
C23A—C22A—H22A	119.00	C23B—C24B—H24B	120.00
C22A—C23A—H23A	120.00	C25B—C24B—H24B	120.00
C24A—C23A—H23A	120.00	C24B—C25B—H25B	120.00
C25A—C24A—H24A	120.00	C26B—C25B—H25B	120.00
C23A—C24A—H24A	120.00	C21B—C26B—H26B	120.00
C24A—C25A—H25A	120.00	C25B—C26B—H26B	120.00
			
C18A—O17A—C14A—C13A	−3.5 (2)	C26A—C21A—C22A—C23A	1.3 (3)
C18A—O17A—C14A—C15A	175.50 (13)	C11A—C21A—C26A—C25A	177.63 (15)
C20A—O19A—C15A—C14A	−174.49 (15)	C22A—C21A—C26A—C25A	−2.4 (2)
C20A—O19A—C15A—C16A	4.3 (2)	C11A—C21A—C22A—C23A	−178.70 (17)
C18B—O17B—C14B—C15B	176.50 (16)	C21A—C22A—C23A—C24A	0.6 (3)
C18B—O17B—C14B—C13B	−3.5 (2)	C22A—C23A—C24A—C25A	−1.4 (3)
C20B—O19B—C15B—C14B	−176.60 (14)	C23A—C24A—C25A—C26A	0.3 (3)
C20B—O19B—C15B—C16B	3.4 (2)	C24A—C25A—C26A—C21A	1.6 (3)
C11A—N10A—C9A—C8A	0.8 (2)	C6B—C1B—C2B—C3B	0.7 (3)
C11A—N10A—C9A—C16A	−178.51 (13)	C2B—C1B—C6B—C5B	−1.7 (3)
C9A—N10A—C11A—C21A	−178.76 (12)	C1B—C2B—C3B—C4B	1.4 (3)
C9A—N10A—C11A—C12A	1.1 (2)	C2B—C3B—C4B—C5B	−2.4 (2)
C11B—N10B—C9B—C16B	178.32 (14)	C2B—C3B—C4B—C7B	174.95 (16)
C9B—N10B—C11B—C12B	0.3 (2)	C3B—C4B—C5B—C6B	1.5 (2)
C11B—N10B—C9B—C8B	−1.4 (2)	C7B—C4B—C5B—C6B	−175.99 (14)
C9B—N10B—C11B—C21B	−179.18 (14)	C3B—C4B—C7B—C8B	58.1 (2)
C6A—C1A—C2A—C3A	−1.2 (3)	C3B—C4B—C7B—C12B	−123.98 (17)
C2A—C1A—C6A—C5A	0.1 (3)	C5B—C4B—C7B—C8B	−124.63 (16)
C1A—C2A—C3A—C4A	1.3 (3)	C5B—C4B—C7B—C12B	53.3 (2)
C2A—C3A—C4A—C5A	−0.3 (2)	C4B—C5B—C6B—C1B	0.6 (2)
C2A—C3A—C4A—C7A	176.64 (15)	C4B—C7B—C8B—C9B	177.54 (14)
C3A—C4A—C5A—C6A	−0.8 (2)	C4B—C7B—C8B—C13B	−0.2 (2)
C7A—C4A—C5A—C6A	−177.73 (14)	C12B—C7B—C8B—C9B	−0.5 (2)
C3A—C4A—C7A—C8A	61.2 (2)	C12B—C7B—C8B—C13B	−178.20 (15)
C3A—C4A—C7A—C12A	−117.36 (16)	C4B—C7B—C12B—C11B	−178.65 (15)
C5A—C4A—C7A—C12A	59.5 (2)	C8B—C7B—C12B—C11B	−0.6 (2)
C5A—C4A—C7A—C8A	−121.89 (15)	C7B—C8B—C9B—N10B	1.5 (2)
C4A—C5A—C6A—C1A	0.9 (3)	C7B—C8B—C9B—C16B	−178.21 (14)
C4A—C7A—C8A—C9A	−175.81 (12)	C13B—C8B—C9B—N10B	179.40 (14)
C4A—C7A—C8A—C13A	5.6 (2)	C13B—C8B—C9B—C16B	−0.3 (2)
C12A—C7A—C8A—C13A	−175.79 (14)	C7B—C8B—C13B—C14B	178.89 (15)
C4A—C7A—C12A—C11A	177.53 (13)	C9B—C8B—C13B—C14B	1.2 (2)
C12A—C7A—C8A—C9A	2.8 (2)	N10B—C9B—C16B—C15B	179.84 (14)
C8A—C7A—C12A—C11A	−1.1 (2)	C8B—C9B—C16B—C15B	−0.4 (2)
C9A—C8A—C13A—C14A	2.8 (2)	N10B—C11B—C12B—C7B	0.7 (2)
C7A—C8A—C9A—N10A	−2.8 (2)	C21B—C11B—C12B—C7B	−179.86 (15)
C7A—C8A—C9A—C16A	176.52 (13)	N10B—C11B—C21B—C22B	24.1 (2)
C13A—C8A—C9A—N10A	175.91 (13)	N10B—C11B—C21B—C26B	−154.66 (16)
C13A—C8A—C9A—C16A	−4.8 (2)	C12B—C11B—C21B—C22B	−155.30 (16)
C7A—C8A—C13A—C14A	−178.60 (14)	C12B—C11B—C21B—C26B	25.9 (2)
C8A—C9A—C16A—C15A	2.1 (2)	C8B—C13B—C14B—O17B	178.65 (15)
N10A—C9A—C16A—C15A	−178.55 (14)	C8B—C13B—C14B—C15B	−1.3 (2)
C21A—C11A—C12A—C7A	178.92 (13)	O17B—C14B—C15B—O19B	0.6 (2)
N10A—C11A—C21A—C22A	29.0 (2)	O17B—C14B—C15B—C16B	−179.42 (14)
N10A—C11A—C21A—C26A	−150.99 (14)	C13B—C14B—C15B—O19B	−179.47 (14)
C12A—C11A—C21A—C22A	−150.90 (15)	C13B—C14B—C15B—C16B	0.5 (2)
C12A—C11A—C21A—C26A	29.1 (2)	O19B—C15B—C16B—C9B	−179.68 (14)
N10A—C11A—C12A—C7A	−1.0 (2)	C14B—C15B—C16B—C9B	0.3 (2)
C8A—C13A—C14A—O17A	−179.21 (14)	C11B—C21B—C22B—C23B	−178.09 (17)
C8A—C13A—C14A—C15A	1.8 (2)	C26B—C21B—C22B—C23B	0.8 (3)
O17A—C14A—C15A—O19A	−4.81 (19)	C11B—C21B—C26B—C25B	178.51 (17)
O17A—C14A—C15A—C16A	176.33 (13)	C22B—C21B—C26B—C25B	−0.3 (3)
C13A—C14A—C15A—O19A	174.25 (13)	C21B—C22B—C23B—C24B	−0.7 (3)
C13A—C14A—C15A—C16A	−4.6 (2)	C22B—C23B—C24B—C25B	0.1 (3)
O19A—C15A—C16A—C9A	−176.15 (14)	C23B—C24B—C25B—C26B	0.4 (3)
C14A—C15A—C16A—C9A	2.6 (2)	C24B—C25B—C26B—C21B	−0.3 (3)

Symmetry codes: (i) −*x*+1, −*y*+1, −*z*+1; (ii) −*x*, −*y*+1, −*z*+1; (iii) −*x*+1, −*y*+2, −*z*; (iv) −*x*, −*y*+2, −*z*+1; (v) −*x*+2, −*y*+2, −*z*; (vi) −*x*+1, −*y*, −*z*+1; (vii) −*x*+1, −*y*+3, −*z*; (viii) −*x*+2, −*y*+1, −*z*; (ix) *x*−1, *y*+1, *z*; (x) *x*, *y*−1, *z*; (xi) *x*+1, *y*−1, *z*; (xii) *x*, *y*+1, *z*.

### Hydrogen-bond geometry (Å, º)

Cg6 and Cg9 are the centroids of the N10*B*,C7*B*–C9*B*,C11*B*,C12*B* and C21*B*–C26*B* rings, respectively.

**Table:**

*D*—H···*A*	*D*—H	H···*A*	*D*···*A*	*D*—H···*A*
C6*A*—H6*A*···O19*B*^iii^	0.93	2.58	3.366 (2)	142
C18*B*—H18*F*···*Cg*9^xii^	0.96	2.93	3.879 (2)	169
C20*B*—H20*D*···*Cg*6^v^	0.96	2.93	3.59 (18)	127

Symmetry codes: (iii) −*x*+1, −*y*+2, −*z*; (v) −*x*+2, −*y*+2, −*z*; (xii) *x*, *y*+1, *z*.
